# Advances in Biomedical Applications of Solution Blow Spinning

**DOI:** 10.3390/ijms241914757

**Published:** 2023-09-29

**Authors:** Javier Carriles, Paul Nguewa, Gustavo González-Gaitano

**Affiliations:** 1 Department of Chemistry, Facultad de Ciencias, University of Navarra, 31080 Pamplona, Spain; jcarriles@alumni.unav.es; 2 ISTUN Instituto de Salud Tropical, Department of Microbiology and Parasitology, University of Navarra, Irunlarrea 1, 31080 Pamplona, Spain

**Keywords:** solution blow spinning, electrospinning, nanofibers, biodegradable polymers, transdermal delivery, tissue engineering, 3D cell culture, regenerative medicine, biosensors, minimally invasive surgery

## Abstract

In recent years, Solution Blow Spinning (SBS) has emerged as a new technology for the production of polymeric, nanocomposite, and ceramic materials in the form of nano and microfibers, with similar features to those achieved by other procedures. The advantages of SBS over other spinning methods are the fast generation of fibers and the simplicity of the experimental setup that opens up the possibility of their on-site production. While producing a large number of nanofibers in a short time is a crucial factor in large-scale manufacturing, in situ generation, for example, in the form of sprayable, multifunctional dressings, capable of releasing embedded active agents on wounded tissue, or their use in operating rooms to prevent hemostasis during surgical interventions, open a wide range of possibilities. The interest in this spinning technology is evident from the growing number of patents issued and articles published over the last few years. Our focus in this review is on the biomedicine-oriented applications of SBS for the production of nanofibers based on the collection of the most relevant scientific papers published to date. Drug delivery, 3D culturing, regenerative medicine, and fabrication of biosensors are some of the areas in which SBS has been explored, most frequently at the proof-of-concept level. The promising results obtained demonstrate the potential of this technology in the biomedical and pharmaceutical fields.

## 1. Introduction

Over the past few decades, nanofibers have attracted attention both in science and industry due to their unique properties and applications [1]. With typical diameters ranging from less than 100 nm to a few micrometers, nanofibers share features with other types of nanosized materials, such as nanoparticles and nanorods. They possess a high surface-to-volume ratio, customizable mechanical properties, and porosity and can be widely functionalized [2]. The broad appeal of nanofibers stems from their production from a wide range of materials, such as synthetic and natural polymers, oxides, and composites, and they exhibit a diverse array of physical properties that can be tailored by altering their surface characteristics [3]. 

Various criteria have been used to classify the methods of production of nanofibers. When their form of production is taken into account, the methods fall within top-down or bottom-up approaches. In bottom-up methods, fibers are produced using their constituting molecules. Examples of these are phase separation, interfacial polymerization, and spinning techniques. In top-down methods, the original material is broken down into fibers by means of physical or chemical treatments [4]. Another classification is based on the type of treatment, either physical or chemical, used for the production of the fibers. Physical approaches introduce energy into the material by mechanical, electrical, thermal, or radiative procedures, and consequently, they are considered top-down techniques. Chemical techniques are usually bottom-up approaches, as they rely on chemical reactions to combine the fiber components [5]. Currently, several reviews describing and comparing the different nanofiber production technologies and their applications, especially electrospinning (ES), have been published [2,3,5,6,7,8,9,10]. However, only a few have focused exclusively on solution blow spinning (SBS) [11,12,13,14], a bottom-up, physical method according to the above classification. In this review, we have attempted to gather the most relevant scientific papers in which SBS methods are utilized for the generation of polymeric fibers, specifically for biomedical applications. In order to facilitate the work of researchers who are new to this technology, a background on the fundamentals of the technique and the variables that affect the quality of fibers is also provided.

## 2. Fundamentals of Solution Blow Spinning (SBS) for Fiber Production

### 2.1. Electrospinning (ES)

SBS shares a number of features with other spinning methods of fiber production, such as ES and melt-blowing (MB). Nowadays, ES is considered the reference method for biomedical applications, and we will first focus in this section on the fundamentals of this technique and the variables that control the morphology of the fibers. We refer the reader to a selection of classical and recent review papers that describe ES and its applications for deeper insight [15,16,17,18,19,20].

From a historical viewpoint, the production of fibers is linked to the understanding of the interactions between electric fields with fluids and drops, with Willian Gilbert being the first to describe the electrostatic attraction of a liquid in the 17th century. A century later, George Mathias Bose improved the static electricity generating machine and, for the first time, aerosols could be generated by applying electric fields to droplets [21]. In the 19th century, Louis Schwabe invented the extrusion spinneret and used it to spin fibers with glass and, in 1887, Charles Vernon Boys developed a device that could produce fibers from various materials such as shellac, beeswax, and collodion [22]. In 1912, John Francis Cooley patented the first ES device and tested his machine by producing nitrocellulose fibers [23]. Twelve years later, Wiegand and Burton conducted studies on the relationship between surface tension, charge, and the effects of electricity on liquid streams and water drops [24]. ES gained popularity as a fiber production technique in the following years. In 1938, Rozenblum and Petrayanov-Sokolov generated carbon-fibrous materials that were used as filters [25]. This led to the manufacture of electro-spun smoke filters for gas masks. Between 1964 and 1969, Geoffrey Ingram Taylor made significant advances in the theoretical fundamentals of ES and developed a mathematical model that explained the cone shape formed at the tip of the spinneret when an electric field is applied [26]. The following years would witness a rapid development of this technique, which has currently become the reference method for the production of micro and nanofibers. 

In ES, the formation of polymeric fibers involves dissolving a polymer in a compatible, volatile solvent (isopropanol, ethanol, acetone, dichloromethane…) in concentrations that vary between 5 and 30% (*w*/*w*). The solution is forced to pass through a thin capillary with a syringe, and an electric voltage (10–40 kV) is applied between the needle tip and the collector that generates charges on the surface of the droplet. The charge repulsion, induced by the electric voltage, opposes the surface tension, and distorts the spherical droplet that becomes cone-shaped (Taylor cone). Once the solution cone is formed, the solution is projected towards the collector as the solvent evaporates during flight, forming thin fibers [27,28].

The parameters that affect fiber morphology depend on the spinning device (electric field, flow rate, needle diameter, and distance between syringe tip and collector), the polymer solution (concentration, viscosity, and solution conductivity), and the environmental conditions (relative humidity and temperature) [29]. Regarding the first factor, fiber diameter usually decreases when increasing the applied voltage, which in turn depends on the type of polymer [30]. The increase in the flow rate leads to homogeneous and bead-free nanofibers. Nevertheless, when the voltage or flow rate exceeds certain critical values, blobs form due to instability in the Taylor cone and/or the incomplete evaporation of the solvent during the time of flight [5,31]. Furthermore, the distance between the needle tip and the collector affects fiber diameter, and more homogenous and thinner fibers can be obtained by increasing the distance as solvent evaporation becomes more efficient [32]. 

Polymer concentration and viscosity play a major role in the fiber production process. By increasing the concentration, the solution becomes more viscous, and this facilitates the entanglement of polymer chains [33]. Solution conductivity also affects nanofiber diameter. Dielectric solutions lack electrical charges that can migrate to the solution–air interface, thus limiting the formation of the Taylor cone that initiates the ES process. This can be overcome with the addition of salts, such as KH_2_PO_4_ or NaCl, to the solution [34,35]. Likewise, the solvents must be carefully chosen. Non-volatile solvents that do not fully evaporate during the flight from the needle tip to the collector tend to produce polymer clusters or large domains of non-fibrous material. On the other hand, if the solvent is excessively volatile, premature evaporation on the tip could lead to the distortion of the Taylor cone or even to the blockage of the nozzle [36]. Environmental variables, such as temperature or chamber humidity, can thus be controlled to optimize solvent evaporation.

The reason ES has become the most important technique in the field of nanofiber production derives from the fact that many materials, not just polymers, can be produced in the form of electro-spun nanofibers, including oxides, ceramics, or carbon composites [37]. In addition, ES allows fiber functionalization before the spinning process by incorporating active substances into the polymer solution or after fiber formation by means of surface modification [38]. In addition, nanofiber production by ES is cheaper compared to other methods, such as fiber extraction. Some representative applications of ES in biomedicine and their associated experimental conditions are summarized in Table 1.

In spite of its widespread use, ES presents some downsides. The most important derives from the requirement of intense electric fields, which limits the range of applications. In general, the typical fiber production rate of ES is low for industrial and commercial purposes (0.1–1.0) g/h [20], and multi-needle and syringeless ES have been developed to overcome this limitation. The first method mounts several nozzles in parallel, each one producing a jet of polymer. The repulsion among charged jets during the spinning may lead to poor-quality fiber and, by covering the multi-jet spinneret with an external electrode, the repulsion between jets is minimized, allowing the distance between needles to be reduced [51]. Needleless ES is based on the immersion of a cylindrical rotor into the spinning solution. A thin layer of polymer solution forms on the surface and, due to the high rotation speed, conical spikes emerge from the surface, producing the Taylor cone upon application of a voltage [52]. More advanced ES techniques have been developed to achieve better control of the composition and morphology of the nanofibers, such as coaxial and triaxial ES, which allow the production of core-shell nanofibers by using a spinneret with up to three coaxial capillaries, through which different polymers are injected [53]. Finally, the need to use organic solvents in ES in most cases entails environmental considerations, although this drawback is common to other methods of production of polymeric fibers.

SBS also shares features with melt blowing (MB), a widely used method to produce fibers, developed in the 1950s at the U.S. Naval Research Laboratory by Wente [54]. Due to the numerous similarities it shares with SBS, it is worthwhile to include here a brief description of this technique. We refer the reader to a selection of articles for those who wish to delve further into the topic [55,56,57,58,59]. In MB, a thermoplastic polymer is melted and passed through a syringe. Subsequently, a hot air stream stretches the polymer jet into fibers, which are deposited on the collector. The morphology of the fibers depends primarily on the polymer flow rate. Increasing the flow rate results in thicker fibers, and their diameter is inversely proportional to the air velocity (working with high pressures can lead to fiber rupture). Finally, by increasing the temperature or the air temperature, the viscosity decreases, and consequently, the diameter of the fibers is reduced [60,61]. Although its primary use is in the production of filters, for water and wastewater filtration, osmosis or chemical filtration in general, MB is solvent-free, and it does not require the use of electric fields.

### 2.2. Solution Blow Spinning (SBS)

**Figure 1:**
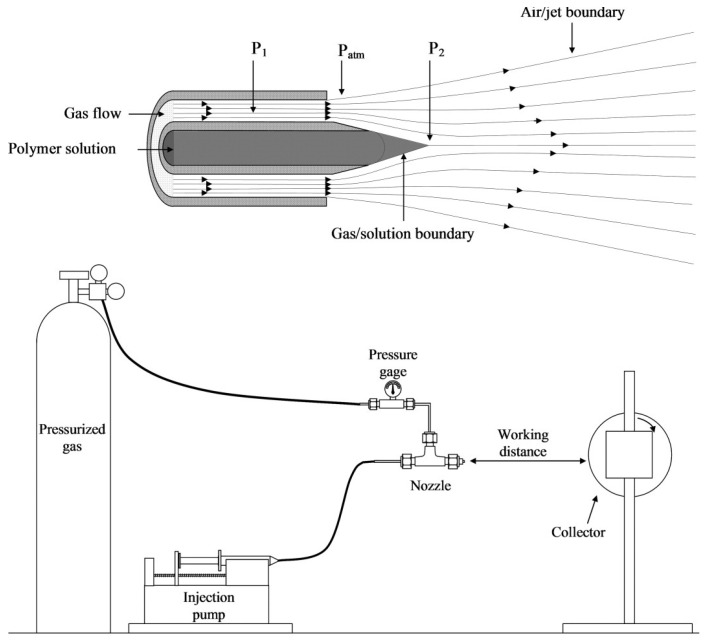
Schematic representation of a typical SBS apparatus (reproduced from reference [13] with permission).

SBS, first described in 2009 by Medeiros [62], can be considered a combination of ES and melt-blowing technology [13]. The typical SBS setup involves a concentric nozzle with two channels (one for the polymer solution and another for the gas stream), a compressed gas source, and a collector [63] (Figure 1). In the SBS process, a region of low pressure is created around the inner nozzle when the gas flows. According to Bernoulli’s principle, the increased velocity of the air in the outer nozzle makes the pressure drop at the nozzle tip [64], shaping the solution into a structure similar to the Taylor cone that forms in the ES process. The pressurized gas stretches the drop formed at the tip of the nozzle, creating an ultrathin jet when the surface tension of the solution is overcome. As the jet travels, the solvent evaporates, and thin fibers are formed and deposited in a collector [65,66].

The main variables that control fiber morphology depend on the solution and the experimental setup (processing) (Table 2) [67,68]. In turn, some variables are interconnected, for example, concentration and viscosity. In what follows, we shall focus on the production of polymeric fibers, which have been almost exclusively investigated to date for biomedical applications.

**Figure 2:**
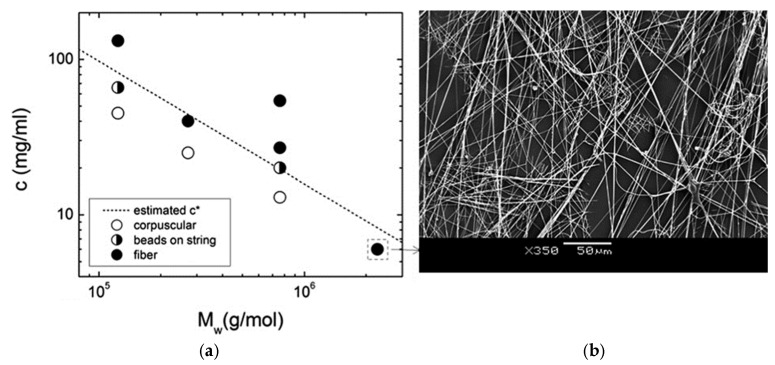
(**a**) Estimated overlapping concentration, c*, for PMMA as a function of the average molar mass, *M_w_*, and polymer concentration, showing the observed fiber morphology, (**b**) SEM images of PMMA fibers (reproduced from reference [71]).

Regarding the solution variables, the concentration is the most important one in order to obtain fibers with a homogeneous morphology [64]. The fiber formation implies the entanglement of polymeric chains, and the key parameter is the overlapping concentration, c*, which marks the threshold between the diluted and semi-diluted regimes. When the polymer concentration surpasses c*, chain overlapping occurs, which favors the formation of continuous fibers. Conversely, when the polymer concentration falls below c*, beaded structures and clusters appear. The extent of chain entanglement depends on both polymer concentration and molecular weight [14,69] (Equations (1) and (2)):

(1) c*=632Mw8NAR232    

(2) $$\langle R^{2}\rangle =\alpha^{2}C_{\infty }(2M_{w}/M_{0})l^{2}\;\;\;\;\;\;$$

where *M_w_* is the average molecular mass, *N_A_* is the Avogadro’s Number, and 〈*R^2^*〉 is the mean-square end-to-end distance of the polymer, which is assumed to be linear. This can be estimated using Equation (2), where α is the Flory expansion factor, *C_∞_* is the characteristic ratio, and *l* is the bond length [69]. As an example, Figure 2 illustrates the correlation between the fiber morphology of PMMA versus concentration and molar mass (c* is shown as a dashed line). When the *c*/c* ratio approaches 1, a transition from bead and corpuscular structures to fiber morphology is observed. A diluted solution of a polymer with a sufficiently large molecular weight can generate bead-free and cluster-free fibers. This observation supports the notion that fiber formation is not solely determined by chain entanglement but also by solution viscoelasticity, as discussed by Yu et al. [70,71].

Fiber morphology is affected not only by solution viscosity but also by surface tension and gas pressure since these parameters control the shear forces in the liquid–air interface. In general, viscous solutions tend to produce thicker fibers [72]. The balance between viscosity and surface tension affects fiber homogeneity, which can be related to the Capillary Number (*Ca*, Equation (3)). The polymer jet is early destabilized for solutions with a low *Ca*, which leads to the formation of beads. On the contrary, decreasing the surface tension while keeping the viscosity constant within moderate *Ca* values permits smoother and more homogenous fibers to be obtained [63].

(3) Ca=μV/γ

As in ES, the solvent plays a critical role in fiber formation by SBS. Oliveira et al. used Hanse parameters to predict the effect of different solvents on the morphology of the blow-spun fibers [73]. According to this scheme, the cohesive energy can be split into polar interactions (*δp*), hydrogen bonding (*δh*), and dispersive van der Waals interactions (*δd*), so materials with similar Hanse parameters are more miscible. Likewise, viscosity and surface tension can be connected to these parameters [74,75]. The presence in the solution of structures other than the polymer, such as nanoparticles, biomolecules, or small molecules in significant proportions, may also modify the physicochemical properties of the solution. Therefore, it becomes essential to determine experimentally the appropriate solvent-polymer combination for a given system.

The volatility of the solvent is a key factor to consider since it determines the evaporation rate. Solvents with a high boiling point tend to produce heterogeneous fibers due to incomplete evaporation during jet flight. If this is the case, the fibers fuse before and after reaching the collector, and wet films are obtained. This is why organic solvents are preferred to water [75,76]. The working distance (nozzle-collector) should then be expanded to ensure the full evaporation of the solvent or the temperature increased by introducing a hot air current [77,78]. In some cases, the average fiber diameter has proven to be more dependent on solvent evaporation rather than on the rheological properties of the polymer solution. 

Pressure and shear differences at the solution–gas interface are responsible for deforming the polymeric solution into a conical shape and further stretching it into an ultrafine fiber when the surface tension is overcome [79]. It has been reported that the type of gas used may affect fiber diameter. Khayet et al. produced polyethersulfone (PS) fibers using different gases [80] and observed a decrease in diameter in the order air < O_2_ < N_2_ < CO_2_ and Ar, with larger pore sizes when Ar or CO_2_ were used. This evidence was explained in terms of the different thermal conductivity of the gases, which would affect the solvent evaporation rate (the higher the thermal conductivity, the faster the evaporation). Gas pressure also influences the quality of the fibers. In general, increasing pressure leads to a more uniform fiber distribution, with a linear correlation between fiber diameter and gas pressure. However, low pressures would not form the cone, while excessive pressure would destabilize it, and the fibers may adopt a curled morphology due to the turbulent flow of the gas [81]. At high pressures, the evaporation rate intensifies, producing thinner and longer fibers, especially when polymer concentration is high [82,83].

The solution flow rate is another relevant parameter to be considered. In the case of commercial airbrushes, the polymer solution enters the nozzle by gravity or by suction. In homemade apparatuses, an automatic syringe can pump the solution in a controlled manner, as in ES. In general, the flow rate should be adjusted to an intermediate value to obtain uniform sub-micrometric fibers: A low feed rate does not supply sufficient solution to produce a stable cone, while high values would release too much, producing agglomeration and incomplete solvent evaporation, with thicker fibers with broad diameter distributions [84,85]. Thus, the optimal value of this parameter should be adjusted considering the other processing variables [64]. In commercial airbrushes, the solution flow is determined by the air pressure, the inner dimensions of the concentric nozzle, and viscosity.

Regarding the type of collector device (static or otherwise), this does not affect per se the fiber morphology. However, if a rotating drum is used, the angular velocity may have an impact on fiber alignment and, as a consequence, on the mechanical properties of the material. Static collectors favor the random orientation of the fibers while rotating collectors produce preferential orientations [86]. Likewise, the distance between the collector and the nozzle does not have a marked impact on fiber morphology or the diameter, but it should be long enough to facilitate the complete evaporation of the solvent (otherwise, polymer clusters, fused fibers, or large polymer domains in the form of films will appear) [87].

As for the relationship between nozzle diameter and fiber diameter, this is not straightforward. In general, the optimal nozzle diameter minimizes fluctuations in the cone shape and permits its stabilization. Typically, a smaller nozzle diameter results in the formation of bead-free fibers or granules. Conversely, for a constant solution feed rate, a larger nozzle diameter tends to yield wider fiber diameters [79]. 

Additionally, environmental conditions can also affect the quality of the resulting material. Temperature plays a crucial role in the volatility of organic solvents, and their evaporation rate can be increased by raising the temperature in the SBS chamber. Conversely, low temperatures can induce bead formation due to incomplete solvent evaporation [88]. Finally, relative humidity has also been proven to modify the porosity of the resulting material, with a considerable presence of micropores on the fiber surface being associated with values of relative humidity exceeding 30% [89,90].

In conclusion, a certain polymer-solvent combination will require optimization of the available experimental setup in order to obtain fibrous materials of the desired quality. The main advantages of SBS over other methods of fiber production, such as ES, derive from the use of simpler instrumentation. The pressurized gas allows the use of non-dielectric solvents and the deposition of the fibers in situ on many different surfaces, including living organs. However, as a relatively new technique, SBS still needs development for its applications at an industrial level. Likewise, although the available “palette” of polymers and solvents that can be used in biomedical applications is limited, the possibility of functionalizing the fibers by incorporating active principles into the solution, for example, drugs, nanoparticles, or biopolymers, involves a great deal of experimentation on the compatibility of the different components of the solution to be blow-spun. 

**Figure 3:**
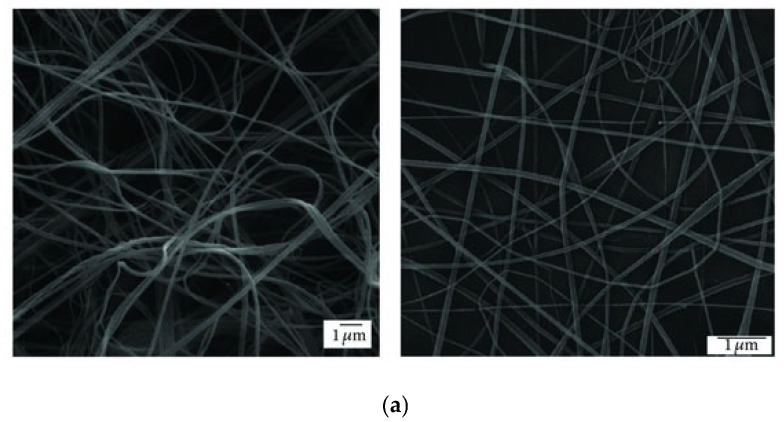

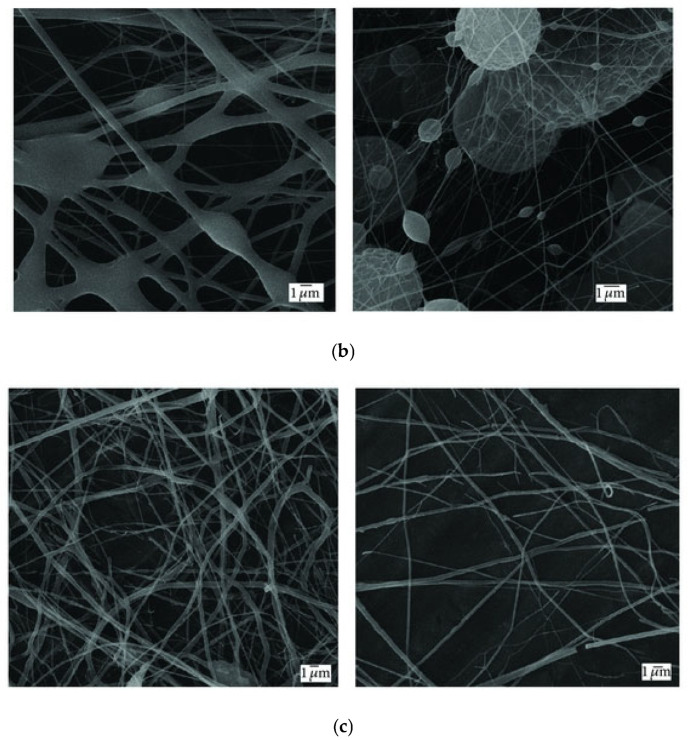
SEM images of (**a**) PLA, (**b**) PCL, and (**c**) PEO fibers produced by ES (**right**) and SBS (**left**) (reproduced from reference [91] with permission).

At this point, it is worth comparing the morphology of the products obtained through SBS and ES. There are a few studies dedicated to this particular aspect. In 2013, Oliveira et al. conducted a comparative study of the fibers produced with three polymers (PLA, PEO, and PCL), yielding distinct results for each (Figure 3) [91]. The PLA and PEO fibers exhibited a more consistent and smooth morphology with both techniques, whereas the PCL fibers obtained through SBS showed a highly irregular structure. In general, there was greater homogeneity in the diameter distribution of matrices produced by ES, with a slightly smaller average diameter, attributed to the turbulence of the gas flow in SBS. Another study conducted by Wojasiński et al. yielded similar results regarding the morphology of PLLA fibers generated by ES and SBS [67]. It must be considered that, at present, ES is a much more developed technique than the relatively novel SBS, and finding the right fit between key variables to produce fibers of good quality for a certain system requires extensive experimental work. However, the growing number of papers dealing with these fundamental aspects point to a fast development of the SBS technology.

## 3. Biomedical Applications of SBS

The current interest in the application of polymeric nanofiber-based materials obtained by SBS in the fields of engineering, textiles, and biomedicine has considerably increased in recent years [11,12,13,14,92]. These materials can be used in the development of electrodes for fuel cells and supercapacitors [93], as sensors to detect organic molecules, gases, or pH [94,95], in membranes for air or liquid filtration [96], photocatalysis [97], and superconductors [98], amongst other examples. In this review, we have focused on those investigations that apply SBS technology to produce fibers, either bare or functionalized, intended specifically for biomedical applications.

### 3.1. Drug Delivery and 3D Cultures

When organs or tissues are damaged, the self-healing response of the body might not efficiently restore normal physiological functions, and the administration of drugs becomes necessary to enhance and accelerate the regenerative process. Orally administered drugs are easy use and may be readily stored and transported, but they may lead to slow absorption rates and poor efficacy for the drug to reach the target area. Administration via injection can be used for a large number of patients when drug toxicity is low, but frequent administrations may be required if adverse effects due to high doses need to be avoided [99,100]. Recently, the use of nanofibers has been found to be an interesting strategy for drug delivery, which has overcome several drawbacks related to other routes of administration since nanofibers can locally accumulate high levels of drug, reducing drug concentration in the systemic circulation and potential adverse reactions or toxic secondary effects [101]. 

Drug-loading technology is a critical step to achieving an optimal release mechanism. The encapsulation method is based on the incorporation of drugs within the polymer, which, in turn, prevents drug degradation. Currently, this is only possible with biodegradable or biocompatible polymers [102]. Chemical immobilization methods are based on the covalent linking between the drug and the nanofiber, which can reduce drug release. Finally, physical adsorption is based on weak intermolecular interactions (hydrogen bonding, van der Waals forces, and hydrophobic effects) between the polymeric fibers and the drug. SBS methods have been used in combination with these different drug-loading strategies, and the first study describing drug release from a polymeric nanofiber was published by Oliveira et al. in 2013 [103]. Table 3 lists the published papers reporting the application of SBS for drug delivery since then.

For comparison purposes between different spinning methods, in a paper published in 2015 by Souza et al. [106], the authors compared the release kinetics of Linalool from PLA fibers produced by ES and SBS. Firstly, it was observed that the morphology of the fibers obtained through both techniques was similar. The PLA fibers produced by ES and SBS had comparable diameters, 176 and 185 nm, respectively. Upon increasing the drug concentration to 20%, the average diameter of SBS fibers remained unchanged (186 nm), whereas that of ES fibers increased (240 nm). In both cases, Linalool was evenly distributed throughout the fibers. With both types of fibers, the release followed a biphasic behavior characterized by an initial slower phase and a second phase marked by faster release. However, significant differences were observed in the release half-time, which was faster in ES fibers (*t*_1/2_ = 575 s) as compared to SBS fibers (*t*_1/2_ = 1645 s).

The possibility of releasing drugs from a polymeric scaffold made of submicrometric fibers can also be used advantageously for the 3D culture of cells and drug screening. Many new drug candidates are synthetized or derived from other precursors to be tested in vitro and in vivo before they can be commercially distributed, and most of them will be rejected because of unexpected toxicity, lack of clinical efficacy, or poor drug properties [120]. Traditionally, the first toxicity and/or activity assays were performed in 2D cultures of well-known cellular lines. Although this is the usual approach, it has limitations in selectivity and sensitivity since 2D cell growth does not completely mimic the genetic profile and cell microenvironment^.^ On the other hand, expensive animal models can reproduce the physiological response of the organism but not necessarily the molecular basis of a specific disease detected in humans [121]. Nanofibers have the scaffolding potential to induce cell growth, migration, and alignment, and they can mimic the extracellular matrix to promote cell–cell or cell–fiber interactions. Furthermore, growth factors or other bioactive molecules can be included in the fibers to promote cell growth or differentiation [122]. These features make 3D cultures a better option than 2D-culture to perform toxicity and activity assays that may help shed some light on the molecular basis of the disease. Here lies the interest of SBS and its versatility to in situ generate 3D scaffolds made of biocompatible submicrometric fibers.

In 2017, Paschoalin et al. investigated cell adhesion and motility using a nanofiber scaffold of PLA/PEG microfibers produced by SBS and analyzed the immunological response to the polymeric fibers [123] by seeding B16F1 and dendritic cells. A clear interaction between both cell types and the scaffold was observed. The remarkable motility detected, a key process in tissue regeneration, was related to a dynamic structure of actin and vinculin proteins at the cell–fiber interface. Immunological detection, performed by analyzing the expression of co-stimulatory proteins (CD80, CD40, CD86) and cytokines (IL-6, IL-10, IL-1β), remained low in the cells seeded. 

Other combinations of biocompatible polymers obtained by SBS have been described. In 2017, Tomecka et al. proposed blow-spun fibers made of PLA and PU as a potential substrate for cardiac cells [124]. Human cardiomyocytes (HCM), grown in polymeric scaffolds, acquired a more elongated shape and arranged in the direction of the fibers. This phenotype and such a special distribution are more similar to cardiac cells in living organisms. Results from proliferation assays showed that HCM grew better in the PLA fibers, and fiber modification with fibronectin, collagen, or laminin reinforced the proliferation ratio. As another example, Molde and co-workers blow-spun fibers over 3D-printed frames with the aim of providing better nutrient transport and cell infiltration into the scaffolds [125]. In this case, tyrosine-derived polycarbonate microfibers were airbrushed iteratively between layers of a 3D-printed support structure. By using this matrix, they observed better cell and vascular infiltration compared to airbrushed fiber mats alone when the scaffolds were implanted subcutaneously.

**Figure 4:**
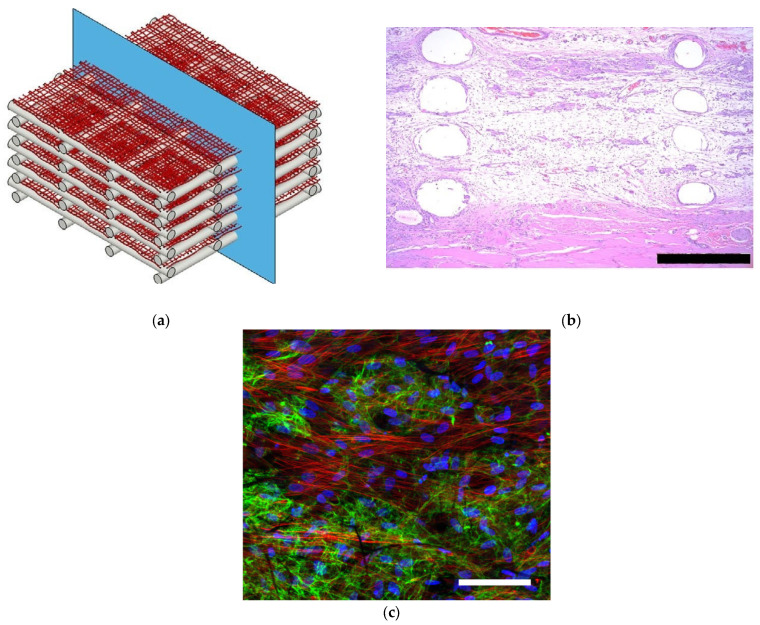
(**a**) Schematic representation of a scaffold made with blow-spun polycarbonate polymeric fibers and 3D-printed frames, (**b**) histological image of the scaffold after being implanted subcutaneously, (scale bars corresponds to 500 µm) (**c**) cellular infiltration and extracellular matrix (ECM) deposited within the scaffold, fibronectin (green), actin (red), and nuclei (blue), (Scale bar correspond to 100 µm) (reproduced from reference [125] with permission).

The production by SBS of 3D scaffolds made of gelatin has also been reported. The scaffolds were used to grow hepatocytes and maintain their function [126] (Figure 4). Hep G2 and primary human hepatocytes showed normal morphology and interactions with the gelatin fibers. Cell viability was not reduced over time, and no toxicity was reported, according to the low expression of the apoptosis marker, caspase-3. The levels of HNF4α and albumin, measured after 24 days, confirmed that the expression of these factors was strong in both cases, indicating a high grade of cell differentiation. In addition, the exposure of the cells to omeprazole, rifampicin, or dimethyl sulfoxide (DMSO) stimulated cytochrome P450 (CYP) expression. The relevance of this investigation lies in the use of a non-synthetic polymer to generate the preforms by SBS. The blow-spun mats showed a 3D fibrous porous structure similar to that of the ECM of mammalian tissue.

More recently, Łopianiak et al. developed non-thrombogenic vascular grafts based on PU by means of a multistep SBS method [127]. The influence of the luminal surface morphology on platelet adhesion and the attachment of endothelial cells was investigated by changing the morphology of the fibers according to the SBS processing parameters (mainly working distance, rotational speed, and polymer concentration). Neither micro nor nanofibers caused hemolysis in contact with blood, and the percentage of platelet-occupied areas for both types of surfaces was comparable to the reference polytetrafluoroethylene (PTFE), used in vascular implants and favored the growth and differentiation of endothelial cells.

### 3.2. Regenerative Medicine and Tissue Engineering

Currently, surgical procedures, transplants (allografts, autografts, or xenografts), and implants are the primary therapeutic options in many disorders. They still present important challenges, such as finding suitable donors, the risk of infections, poor integration of the implant or transplant, and the potential rejection of the graft [128]. The goal of regenerative medicine is to create functional tissues that aid organ repair. This involves utilizing various types of cells (such as stem and mesenchymal cells) and scaffold materials that mimic the cellular microenvironment. Polymeric scaffolds made of nano and microfibers facilitate cell attachment, migration, growth, and cell differentiation [129], and they can be deposited “in situ” on biological substrates [130]. The potential of SBS to generate these scaffolds in situ is evident and represents a yet-to-be-explored avenue in the field of regenerative medicine.

#### 3.2.1. Bone and Cartilage Regeneration

The osteogenic process starts by transforming the cartilage into immature osseous tissue, which will eventually be remodeled by metalloproteinases and osteoclasts to form a robust and well-organized bone [131,132]. Bone tissue has a considerably high regeneration ratio, but it is still a challenge in patients with advanced age or traumas and malignancies. Autografts may cause significant bone loss at the donor site, leading to breakage and morphological mismatch, while xenografts and allografts may have immunological issues due to the degradation of the implant [133]. In this context, Popkov et al. have described a functionalized PLA scaffold obtained by SBS for bone tissue engineering [134]. One of the limitations of the use of polymers as osteo-implants is their low activity in promoting the differentiation of the mesenchymal cells into osteoblasts. This process can be promoted by incorporating hydroxyapatite (HAp) into the fiber scaffold. HAp enhanced the osteogenic properties of the material by improving osteoblast viability and raising osteocalcin alkaline phosphatase and RUNX2 levels without toxicity. The increase in the HAp content did not modify the phase composition and wettability of the scaffold, although the uniaxial strength and relative elongation were reduced. The SBS scaffolds were implanted in the parietal bone of a rat’s skull, which promoted angiogenesis, and it was progressively replaced by its own tissues in the range of 60 to 90 days. The osteogenic properties of the HAp scaffolds were not only improved by the inorganic particles but also by the larger diameters and porosity, which contributed to cell migration and infiltration and to nutrient diffusion. The blow-spun scaffold could thus be appropriate for replacing bone defects, particularly in long tubular bones, according to the authors. These results support those of Liu. et al., who studied the same PLA-HAp system for its application in bone tissue repair and concluded that this material favored cell proliferation and alkaline phosphatase activity. The effectiveness of the SBS scaffolds was found to be comparable to that produced by ES [135]. 

Regarding the regeneration of cartilage tissue, this is a complex process in which the regenerated tissue does not usually present the original features, mainly due to low vascularization and innervation. In addition, the dense ECM limits chondrocyte migration and nutrient diffusion towards the injured area [136]. Conventional treatments (osteochondral autografts or allografts) show limited therapeutic efficacy in many cases and may also lead to post-traumatic osteoarthritis [137]. The development of engineered cartilage usually combines cells with different kinds of natural or synthetic scaffolds, but this is not easy either due to the unique organization of collagen in the cartilage tissue [138]. In a proof-of-concept study, Dorthe et al. produced collagen scaffolds by SBS, which were treated with glutaraldehyde to facilitate crosslinking [139]. The scaffolds were seeded with IPFP and, after three weeks, a fibrocartilage neotissue appeared, and an ECM formed by glycosaminoglycans and collagen I and II. Finally, these scaffolds were used to treat defects in bovine meniscus and osteoarthritic human meniscus. After three weeks, and as in the in vitro assays, fibrocartilaginous tissue was developed, with an ECM containing the glycosaminoglycans present in the host tissue. Although enhanced mechanical properties need to be optimized for translation to clinical application, the results are promising and show that SBS can rapidly generate collagen fibers for cartilage regeneration and engineering. The authors also discussed the use of electro-spun or blow-spun fibers, highlighting that the materials produced by ES have a higher density and lower porosity, which, in this case, may limit cell growth and cell infiltration into the implant.

#### 3.2.2. Skin Regeneration

A functional biomaterial for skin regeneration constitutes a barrier against infective agents, providing sufficient mechanical strength to protect the wound and mimicking the ECM. Tien et al. developed transdermal blow-spun patches with chitosan for dermal wound healing [140]. This polymer presents some interesting properties (non-toxicity, biocompatibility, and biodegradability), but perhaps the most relevant for this application is its bacteriostatic activity. The positive charges of the amino group can interact with the negative charges from the bacterial cell wall, disrupting the membrane, altering its permeability, and inhibiting DNA replication [141]. In addition, chitosan is a hemostatic agent [142]. In this study, in vitro assays performed with fibroblasts (NHDF line) demonstrated no toxicity, with a flattened morphology of the fibroblasts. They also secreted cytokines involved in the wound healing process (IL-6 and IL-8, which play a key role in acute inflammation), HGF, which stimulates endothelial and epithelial cell proliferation, and MCP-1, involved in the recruitment of monocytes [143,144]. Similar results have also been reported supporting the use of transdermal patches of chitosan/PEO fibers produced by SBS, in this case, with the scaffold modified with PDS and with electro-spun chitosan/PEO fibers. In both cases, it was demonstrated that the material was not toxic and favored cell growth. Although the healing tests were performed with different methodologies in both experiments, it was shown that the blow-spun fibers significantly contributed to cell migration and the healing process [145,146].

**Figure 5:**
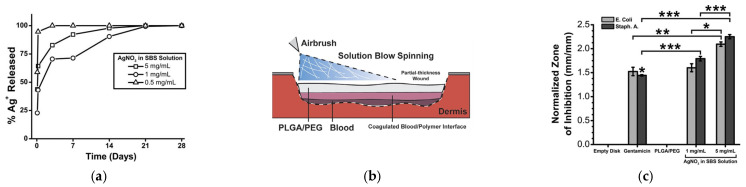

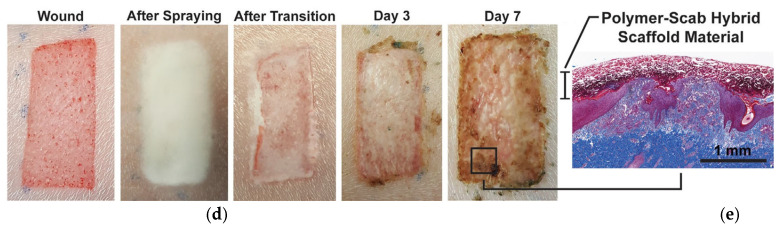
Silver-functionalized blend fibers of PLGA/PEG obtained by SBS and tested on a porcine wound model. (**a**) Fraction of silver ions released over time, (**b**) Schematic representation of the in situ wound dressing produced by SBS; (**c**) Zone of inhibition produced by PLGA/PEG as a function of the silver content, * *p* < 0.05; ** *p* < 0.01; *** *p* < 0.001; (**d**) wound progression before and after the wound dressing was applied; (**e**) histological cross-section of wound biopsy after seven days (reproduced from reference [147] with permission).

In 2020, a sprayable wound dressing exhibiting antimicrobial properties was reported. The blow-spun dressing contained blend fibers of biodegradable and absorbable PLGA and PEG, and AgNO_3_ as an antimicrobial agent [147]. The morphology, thermal, and mechanical properties were fully characterized, as well as the silver release, to evaluate the feasibility of the procedure. In vitro studies showed that these dressings had low cytotoxicity and antimicrobial activity and achieved silver controllably released over 7–14 days. The dressings were also tested in vivo on a porcine partial-thickness wound model by creating the wound and spraying the fibers directly with an airbrush. The PLGA/PEG was incorporated into the wound, forming a durable scaffold and barrier throughout the re-epithelialization process (Figure 5). Beneficial features were observed: greater exudate absorption, integration into the wound, and tissue regeneration with higher vascularization. The authors concluded that the sprayed dressings, both silver-functionalized and non-antimicrobial, were effective, without causing significant complications or delays in healing in the porcine wound model used, with healing rates similar to those of clinical PU dressings, with similar scarring.

#### 3.2.3. Soft Tissue and Vascular Regeneration

In some disorders, such as bowel obstruction, the adjoining of healthy edges of ischemic tissue is required. In many cases, after the surgery, deposition of connective tissue in the surgical area may occur, with subsequent damage to the serous tissue. An optimal adhesion tissue must help prevent the deposition of inflammatory scar tissue. Apolipoproteins (ApoE) are involved in cholesterol and fatty acid transport and present anti-inflammatory properties in intestinal disorders [148,149]. ApoE functionalized fibers can be used to address these drawbacks. Metecan et al. developed a biocompatible sealant based on blow-spun PLCL functionalized with an ApoE-derived peptide (COG133). Cecal ligation and cecal anastomosis were performed on C57BL/6 female mice. The experiments showed that the surgical sealant significantly reduced the formation of postoperative abdominal adhesions. A reduction in the anti-inflammatory cytokines, as a result of COG133 modulation, was also reported [150].

Surgical sealants designed for internal use often face issues of inadequate adhesion or the potential for cytotoxic effects. Recently, a study suggested applying a novel surgical sealant derived from a polymer blend comprising poly(lactic-co-glycolic acid) (PLGA) and poly(ethylene glycol) (PEG) [151]. The innovation highlighted the integration of nano-to-microscale silica particles, enhancing adherence to wet tissue while maintaining cell viability, biodegradation rate, and minimizing local inflammation.

Regarding the regeneration of vascular tissues, the low availability of suitable tissue in advanced-aged patients for the replacement of small with autologous vessels represents a hurdle. For these patients, the manufacture of artificial vessels has shown major disadvantages due to compatibility issues, poor mechanical properties, and inadequate cell proliferation in the vessel that may cause thrombotic episodes or intimal hyperplasia. Due to their low thrombogenicity, polyurethane (PU) scaffolds based on nanofibers have been proposed as an alternative to overcome the limitations of synthetic vessels, as they promote cell proliferation and attachment. In 2021, Kopec et al. designed PU fibrous scaffolds prepared by SBS, coated with polydopamine (PDA) and gelatin, to improve the polymer compatibility with endothelial cells and their hydrophilicity [152]. By coating tubular PU scaffolds and commercial ePTFE with PDA or PDA/Gel, the proliferation of endothelial cells was enhanced, as well as the mechanical properties of the material. Albeit at a proof-of-concept level, the approach proved efficient in improving scaffold colonization with endothelial cells, which could work equally upon implantation of a blow-spun polymeric scaffold.

Another approach to designing vascular grafts was developed in 2019 [153]. This new strategy combines a dip-spinning technique for applying concentric layers of cell-laden hydrogels, along with a modified solution blow spinning (SBS) device for incorporating aligned nanofibers as reinforcement. The central and outer layers were adjusted to replicate the characteristics of the media and adventitia layers found in native arteries. This enables the fabrication of small grafts that demonstrate mechanical responses and compliance similar to the J-shape observed in human coronary arteries. The dip-spinning-SBS technology generates constructs with mechanical properties reminiscent of natural tissues and cellular activities derived from cells, which are essential for applications involving clinical bypass procedures.

### 3.3. Other Applications of SBS in Life Sciences

Early diagnosis plays a critical role in the treatment of patients. Accordingly, important research has been carried out to understand illness physiopathology and to develop effective and sensitive methods for the detection of the associated biomarkers [154]. The properties of nanofibers (large surface/volume ratio, porosity, and wide range of compositions) have been exploited to devise sensors capable of analyzing different biochemical markers [155], such as those associated with cancer, cardiovascular diseases, and diabetes, among others [156,157,158]. Most of the publications on this topic describe the use of electro-spun fibers, which are produced with metal oxides, and the output measured by electrochemical and fluorometric methods. The first step in any biosensing process is the biomarker diffusion to the electrode, where the recognition is produced. Then they react with a metal oxide or a biomolecule previously immobilized in the nanofiber [159]. Based on this concept, Li and co-workers developed an immunoassay for the electrochemical measurement of α-fetoprotein (AFP), a biomarker for hepatocarcinoma [160]. The method resulted faster than the conventional ELISA and allowed reducing the analysis time from 24 to 1 h. Other examples of analytical methods are graphene and TiO_2_ nanofibers for ErbB2 (Erythroblastic oncogene B-2) detection in breast cancer [161], PS nanofibers embedded in carboxylated multi-walled carbon nanotubes to detect troponin I in cardiovascular disease [162], and ploly(vinylidenefluoride) and polyurethane nanofibers as glucose sensors in diabetes patients [163,164].

Compared to ES, the number of studies in which SBS is used for the design of biosensors is scarce. The first investigation dates back to 2012, by Oliveira et al. [165]. Blow-spun PLA fibers containing multi-walled carbon nanotubes (MWCNT) were deposited on modified-ITO electrodes, and then the enzyme glucose oxidase (GOD) was immobilized on the fibers by drop coating. GOD catalyzes the conversion of β-D-glucose to D-glucono-δ-lactone in the presence of O_2_, and in this process, the H_2_O_2_ formed is detected by the electrode. The sensor showed a sensitivity of 358 nA/mM and a detection limit of 0.08 mM, which are among the best-reported values up to that date. SBS has also been used to produce immunosensors to detect p53, which plays a major role in the suppression of tumor growth [166]. The biosensors were made with a matrix of PLA fibers blow-spun on gold electrodes. Then, an anti-p53 active layer was immobilized on the fibers by activation of the carboxylic groups of the polymer and incubation with 1-ethyl-3-(3-dimethylaminopropyl) carbodiimide and N-hydroxysuccinimide. The limit of detection of the method was 11 pg/mL, as measured by electrical impedance spectroscopy, which would allow the detection of the p53 biomarker in patient samples. The straightforward procedure of fabrication has a clear scaling-up potential and could be extended to other biomarkers.

Finally, another interesting application of nanofibers obtained by SBS, is the manufacturing of organ-on-a-chip devices (OOCs). OOCs are microfluidic devices designed to mimic certain aspects of human physiology, including biofluid flow, nutrient supply, drug delivery, and oxygen transport. Many cell types not only require 3D scaffolds to express their own phenotype, but they also rely on mechanical stimulation. By incorporating polymeric scaffolds into these devices, it is possible to replicate more accurately the physiological conditions. The scaffold provides structural support for cell growth, while the microfluidic system provides nutrients, drugs, or oxygen gradients necessary for cell functions [167,168]. In 2020, Kobuszewska et al. designed an OOC device based on blow-spun PLLA and PU nanofibers in order to develop a model of hypoxic myocardial tissue for heart regeneration studies under flow conditions [169]. Nanofibrous mats with aligned fibers were produced by SBS. The preferential fiber alignment was achieved by varying the rotational speed of the collector to create a parallel orientation of two different cardiac cell lines, and the cultures were studied by quantitative determination of ATP by bioluminescence under hypoxia conditions. This investigation represents the first study of hypoxia simulation in a microfluidic system in which cardiac cells were cultured on nanofiber mats. 

## 4. Conclusions and Future Directions

In this review, we have attempted to bring together the published works on biomedical applications of SBS, an emerging technology for the production of polymeric micro and nanofibers. As in other methods, the precise experimental conditions to generate the fibers, such as type of polymer, solvent composition, injection rate, pressure, or temperature, have to be optimized for a given application to produce homogeneous morphologies. Although some studies have addressed this matter in the case of biocompatible polymers, the functionalization and their in-service performance for biomedical applications are still at an early stage, with the first investigation in the field dating back to 2013, and on most occasions, at a proof-of-concept level. Fundamental work, both theoretical and experimental, is still necessary to correlate the typical working variables used in SBS with the morphology of the material and the in-service performance.

Blow-spun polymeric matrices have a large surface-to-volume ratio, high porosity, and pore-size distributions that can mimic the ECM. Consequently, the investigations in the literature have been aimed mainly at the production of scaffolds for 3D cell culture and in regenerative medicine and tissue engineering for bone, skin, soft tissue, or vascular regeneration. Other applications, such as the use of blow-spun nanofiber mats as wound dressings, in which a grid of biodegradable nanofibers can facilitate the exchange of gases and liquids with the environment while avoiding infections and facilitating the regeneration of the tissue, or for transdermal drug delivery from functionalized fibers in the form of sprayed patches, have been little explored as yet, despite their evident interest. The versatility of SBS permits the design of portable, hand-held, spinning devices that could be used for in-situ applications to spray fibers on an organ or an incision, acting as a sealant or as a physical barrier to prevent hemostasis during surgical interventions. On the other hand, the design of SBS apparatuses capable of generating ex-situ fibrous biomaterials for large-scale production still requires research and up-scaling if good control of the morphology and the in-service properties of the materials is to be achieved. The combination of SBS with other methods, such as 3D printing technology, is also an incipient and appealing field that still needs development, as well as the design of apparatuses for the production of more complex structures, such as core-shell fibers made of different polymers, for example.

Although at an early stage, we anticipate that the SBS technology will see an expansion over the coming years, especially for biomedical and pharmaceutical applications, both in the knowledge of the conditions to produce the fibers for a specific purpose, as in the design and up-scaling of new SBS devices that permit better control of their morphology and functionality.

## Figures and Tables

**Table 1:** Typical materials and solvents used for the production of electro-spun nanofibers for biomedical applications. TFEA (2,2,2-trifluoroethanol), DCM (Dichloromethane), DMF (Dimethylformamide), PELA (Copolymer PEG and lactide), SF (Silk fibroin), rGO (reduced graphene oxide), RA (retinoic acid), FSG (fish skin gelatin).

Material	Solvent	Application	Reference
PLCL 8%/COL 7.5%	TFEA/DCM (1:1 *v*/*v*)	Tissue regeneration (blood vessels)	[39]
PVA 10%, Chitosan 3%	Water	Wound dressing	[40]
PCL:Gelatin (70:30) 15%	Acetic acid: formic acid (9:1)	Tissue regeneration (neural stem cell differentiation)	[41]
PCL 20%	DCM:DMF (7:3)	Tissue regeneration (bone regeneration)	[42]
PDLA (20–40%)	DMF	Tissue regeneration (post-surgery abdominal adhesions)	[43]
PCL/chitosan 12%	DCM:DMF (4:1)	Tissue regeneration	[44]
PELA 15–30%	DMF:acetone (80:20)	Tissue regeneration	[45]
SF 10%-rGO 3.5%	Acid formic 10% *w/v*	Tissue regeneration (neural differentiation)	[46]
PLGA 20%	Acetone	Tissue regeneration (skin)	[47]
PCL 15%	DMF	Drug delivery	[48]
PCL 15%-RA-CeO_2_	DCM:Methanol (4:1)	Drug delivery (cancer)	[49]
FSG (30%)	Acetic acid 30% (*v/v*)	Drug delivery (food packing)	[50]

**Table 2:** Main variables that affect the fiber morphology in SBS.

Parameters	Effect
Polymer solution	
↑ Polymer concentration	↑ diameter
↑ Viscosity	↑ diameter
↓ Surface tension	↑ fiber homogeneity
↑ Solvent volatility	↑ porosity of fibers
SBS device setup/processing	
↑ Flow rate	↑ diameter
↑ Gas pressure	↓ diameter
↓ Nozzle diameter	↓ diameter
↑ Nozzle-collector distance	↑ fiber homogeneity

**Table 3:** Drug delivery applications that use nanofibers produced by SBS.

Polymer	Drug Loading Technique	Drug	Reference
PLA/PVP	Physical adsorption	Copaiba	[104]
PLA	Physical adsorption	Carvacrol/Tetracycline hydrochloride	[105]
PLA	Physical adsorption	Linalool	[106]
FSG	Physical adsorption	Cinnamaldehyde	[107]
FSG	Encapsulation	Carvacrol	[108]
PVP/PCL	Encapsulation	Sulforhodamine B	[109]
PVP	Physical adsorption	TiO_22_	[110]
PLA/PEG	Encapsulation	Amphotericin B	[111]
PLA	Physical adsorption	Oils from *Ocimun basilicum*	[112]
PLA	Encapsulation	Oils from *Alpinia speciose* and *Cymbopogon glesuosus*	[113]
Chitosan/PCL	Physical adsorption	Thymol/hydroxypropyl-β-cyclodextrin	[114]
PLA/PEG	Encapsulation	Acyclovir	[115]
PLA	Physical adsorption	Oils from *Ocimun basilicum* and *Ocimum gratissimum*	[116]
PEO/chitosan	Physical adsorption	Tenofovir disoproxil fumarate	[117]
PLA	Physical adsorption	Oils from *A. speciosa* and *C. flexuosus*	[118]
PLA	Physical adsorption	Carvacrol and Chlorhexidine	[119]

## Data Availability

Not applicable.

## Abbreviations

COL	collagen
ECM	extracellular matrix
ES	electrospinning
FSG	fish skin gelatin
OOC	organ-on-a-chip
PCL	polycaprolactone
PDLA	poly(D-lactic acid)
PDS	polydimethylsiloxane
PdTFPP	Pd(II) meso-Tetra(pentafluorophenyl)porphine
PELA	polylactide-polyethylene glycol copolymer
PES	polysulfone
PLA	polylactic acid
PLGA	poly(lactic-co-glycolic acid)
PLLA	poly (l-lactic acid)
PMMA	polymethyl methacrylate
PTFE	polytetrafluoroethylene
PU	polyurethane
PVA	polyvinyl acetate
PVP	polyvinylpyrrolidone
SBS	solution blow spinning
SF	silk fibroin
